# Crystal structure of a di­aryl carbonate: 1,3-phenyl­ene bis­(phenyl carbonate)

**DOI:** 10.1107/S2056989017016772

**Published:** 2017-11-28

**Authors:** Marina A. Solomos, Jeffery A. Bertke, Jennifer A. Swift

**Affiliations:** a Department of Chemistry, Georgetown University, 37th and O Sts NW, Washington, DC, 20057, USA

**Keywords:** crystal structure, di­aryl­carbonates, di­phenyl­carbonate, alcoholysis, offset π–π inter­actions, weak C—H⋯O inter­actions

## Abstract

The whole mol­ecule of the title compound, C_20_H_14_O_6_, is generated by mirror symmetry, the mirror bis­ecting the central benzene ring. The carbonate groups adopt an *s-cis-s-cis* conformation, with torsion angles of 58.7 (2) and 116.32 (15)°. The crystal structure of 1,3-phenyl­ene bis­(phenyl carbonate) contains no strong hydrogen bonds, though weak C—H⋯O and offset π–π inter­actions are observed, forming layers parallel to the *ac* plane.

## Abstract 2

The whole mol­ecule of the title di­aryl­carbonate is generated by mirror symmetry, the mirror bis­ecting the central benzene ring, and the carbonate groups adopt an *s-cis-s-cis* conformation. In the crystal, there are only weak C—H⋯O hydrogen bonds and offset π–π inter­actions present.

## Chemical context   

Organic carbonates have a wide range of applications as polymers, surfactants, fuel additives, solvents for complex industrial syntheses and extractions, and even medical agents, dyes, and foodstuff (Shukla & Srivastava, 2017 ▸). They are commonly synthesized by treating alcohols with phosgene, a rather toxic reagent. Alternative preparatory methods include the reaction of alcohols and carbon monoxide in the presence of a catalyst, direct condensation of alcohols and carbon dioxide (Joe *et al.*, 2012 ▸; Zhang *et al.* 2012 ▸; Zhao *et al.*, 2009 ▸), or the alcoholysis of urea (Ball *et al.*, 1980 ▸; Bhanage *et al.*, 2003 ▸; Zhang *et al.*, 2016 ▸; Mote & Ranade, 2017 ▸).

The bis­(phenyl carbonate) structure reported herein was identified as an unexpected side product from the attempted recrystallization of 1-(*m*-phenol)-3-phenyl­urea from ethanol. We surmise this compound formed through a combination of inter­molecular ‘self-alcoholysis’ reactions leading to a carb­amate inter­mediate (Mote & Ranade, 2017 ▸), which subsequently over time yields the title compound, 1,3-phenyl­ene bis­(phenyl carbonate). Compared to the one-dimensional hydrogen-bonded chain motif so frequently seen in di­aryl­urea crystals (Solomos *et al.*, 2017 ▸; Capacci-Daniel *et al.*, 2010 ▸, 2015 ▸, 2016 ▸), di­aryl carbonates lack the ability to associate *via* strong inter­molecular hydrogen bonds. Analysis of the relatively limited number of di­aryl carbonate structures previously reported shows that the title compound shares some of the same structural features.
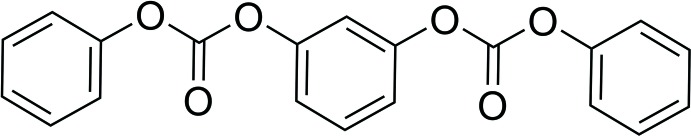


## Structural commentary   

**Figure 1:**
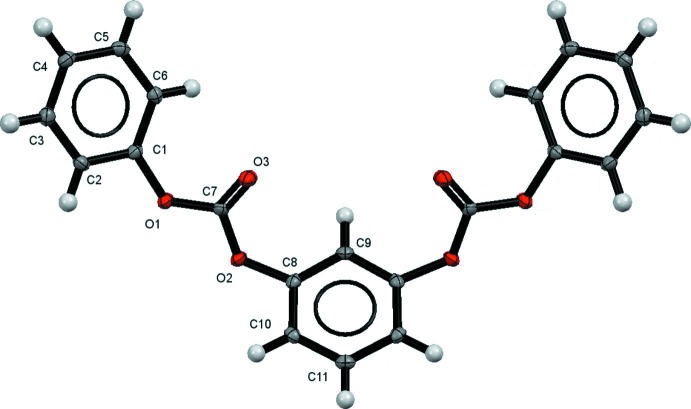
Mol­ecular structure of the title compound, with atom labeling. Displacement ellipsoids are drawn at the 50% probability level. Unlabeled atoms are related to the labeled atoms by mirror symmetry (symmetry operation: *x*, −*y* + 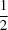, *z*).

The mol­ecular structure of the title compound is shown in Fig. 1 ▸. The asymmetric unit consists of half a mol­ecule, as atoms C9 and C11 sit on a mirror plane. The C7=O3 bond distance [1.1878 (18) Å] and the C7—O1 and C7—O2 bond distances [1.3446 (18) Å and 1.3442 (18) Å, respectively] are in good agreement with values reported for other carbonate structures (Cambridge Structural Database: Version 5.38, Groom *et al.*, 2016 ▸). The aromatic rings are both *s-cis* to the carbonate group with C7—O1—C1—C6 and C7—O2—C8—C10 torsion angles of 58.7 (2) and 116.32 (15)°, respectively. The 1,3-substitution of the central aromatic ring imparts the mol­ecule with a bent or ‘U-shape’ conformation and a significant net dipole moment.

## Supra­molecular Features   

**Figure 2:**
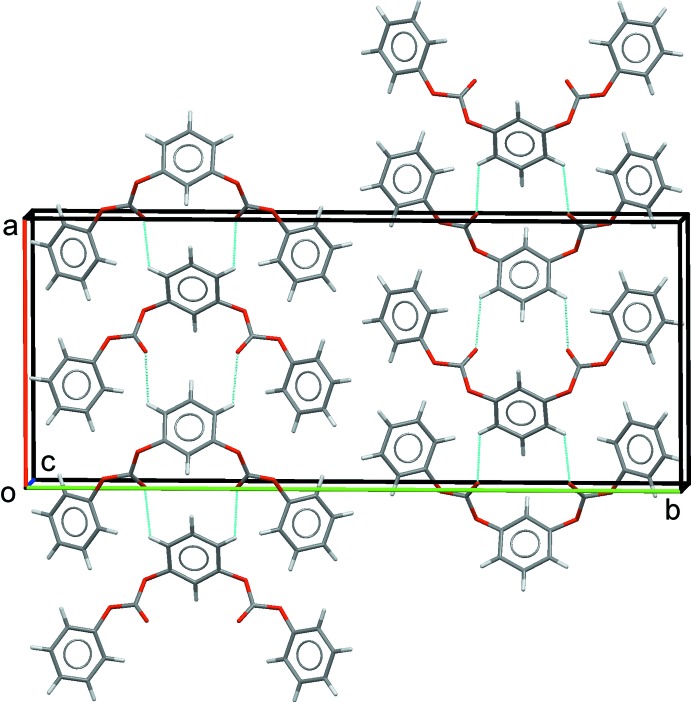
Crystal packing of the title compound viewed along the *c* axis, showing the anti­parallel alignment of adjacent rows of mol­ecules, which creates a long *b* axis of 31.548 (3) Å. The C—H⋯O hydrogen bonds (see Table 1 ▸) are shown as dashed line.

**Figure 3:**
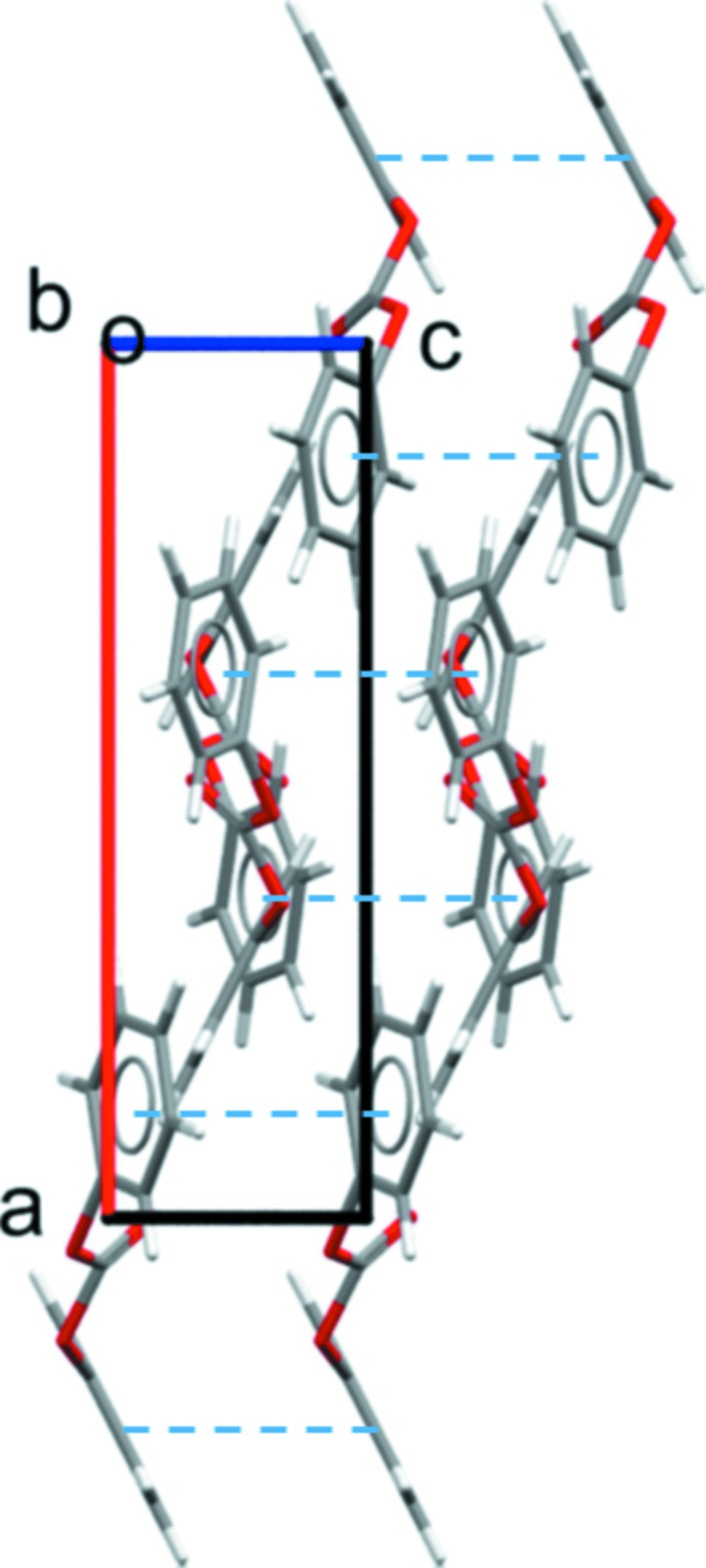
A partial view along the long *b* axis of 31.548 (3) Å of the crystal packing of the title compound, showing the π–π stacking inter­actions (dashed lines).

The lengths of the unit-cell axes in the 1,3-phenyl­ene bis­(phenyl carbonate) structure are strikingly different. Mol­ecules along the *a*-axis direction are related by glide symmetry and assemble into polar chains (Fig. 2 ▸). A short inter­molecular C=O⋯H—C contact (2.59 Å; see Table 1 ▸) between mol­ecules along this axis may favorably contribute to their assembly. The dipoles of adjacent chains in the *ab* plane adopt an anti­parallel alignment, which leads to the very long *b* axis. The very short *c* axis reflects the offset π–π stacking between mol­ecules that are related by translation (Fig. 3 ▸). Details: *Cg*1⋯*Cg*1^i,ii^ = 3.822 (1) Å, inter­planar distance = 3.438 (1) Å, with a slippage of 1.669 Å [*Cg*1 is the centroid of the phenyl ring C1–C6, symmetry codes: (i) *x*, *y*, *z* − 1; (ii) *x*, *y*, *z* + 1]; *Cg*2⋯*Cg*2^iii,iv^ = 3.822 (1) Å, inter­planar distance = 3.398 (1) Å, with a slippage of 1.749 Å [*Cg*2 is the centroid of the central benzene ring, symmetry codes: (iii) *x*, −*y* + 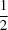, *z* − 1; (iv) *x*, −*y* + 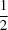, *z* + 1).

## Database Survey   

A search of the Cambridge Structural Database (CSD, Version 5.38 with May 2017 update: Groom *et al.*, 2016 ▸) for organic di­phenyl carbonates yielded 20 hits. Inter­estingly, most of the structures have unit-cell parameters with at least one considerably long axis. With a *b*-axis length of 31.548 (3) Å, the structure of 1,3-phenyl­ene bis­(phenyl carbonate) is consistent with this trend. Across the 20 structures, the C=O bond lengths range between 1.155 and 1.207 Å [average: 1.178 (11) Å], C—O bond lengths fall within 1.310 and 1.387 Å [average: 1.343 (9) Å], and O—C—O angles average 106 (1)°. However, torsion angles about the C—O—C—C_arom_ bonds are extremely variable.

Only one other acyclic bis­(phenyl carbonate) was identified in this search, 4,4′-iso­propyl­idenediphenyl-bis­(phenyl­carbonate) (DINWOM10; Perez & Scaringe, 1987 ▸). The bond lengths and angles are in good agreement with our structure, with C=O = 1.152 and 1.173 Å; C—O = 1.326–1.337 Å and O—C—O = 106.6 and 105.5°. Also similar is the structure of diphenyl carbonate (ZZZPCA02; Hosten & Betz, 2014 ▸), with C=O = 1.188 Å; C—O = 1.343 and 1.337 Å; O—C—O = 104.85°. The aromatic torsion angles for diphenyl carbonate are also similar to the title compound, with C—O—C—C angles of 59.90 and 132.36°.

## Synthesis and crystallization   

Equimolar amounts of 3-amino­phenol and phenyl iso­cyanate were added to benzene under nitro­gen and stirred for 24 h. A white precipitate identified as 1-(*m*-phenol)-3-phenyl­urea was filtered, dried, and recrystallized in assorted organic solvents (ethanol, methanol, acetone, ethyl acetate, benzene, toluene, acetone:hexa­nes, aceto­nitrile). Slow evaporation of an ethano­lic solution in a 1 dram vial, capped with pierced lids, yielded large colorless plates of 1,3-phenyl­ene bis­(phenyl carbonate). Needle-like crystals identified within the same vials corresponded to 1-(*m*-phenol)-3-phenyl­urea. The appearance of 1,3-phenyl­ene bis­(phenyl carbonate) crystals was not consistent across multiple recrystallization experiments, suggesting that select impurities and/or longer, delayed evaporation methods that favor non-equilibrium products may be needed to obtain this material.

## Refinement   

Crystal data, data collection and structure refinement details are summarized in Table 2 ▸. H atoms were included as riding idealized contributors with C—H = 0.95 Å and *U*_iso_(H) = 1.2*U*_eq_(C).

## Supplementary Material

Crystal structure: contains datablock(s) I, global. DOI: 10.1107/S2056989017016772/su5407sup1.cif

Structure factors: contains datablock(s) I. DOI: 10.1107/S2056989017016772/su5407Isup2.hkl

Supporting information file. DOI: 10.1107/S2056989017016772/su5407Isup3.cml

CCDC reference: 1586885

Additional supporting information:  crystallographic information; 3D view; checkCIF report

## Figures and Tables

**Table 1:** Hydrogen-bond geometry (Å, °)

*D*—H⋯*A*	*D*—H	H⋯*A*	*D*⋯*A*	*D*—H⋯*A*
C10—H10⋯O3^i^	0.95	2.59	3.2105 (8)	123

Symmetry code: (i) 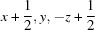.

**Table 2:** Experimental details

Crystal data	
Chemical formula	C_20_H_14_O_6_
*M*_r_	350.31
Crystal system, space group	Orthorhombic, *P* *n* *m* *a*
Temperature (K)	100
*a*, *b*, *c* (Å)	12.9597 (12), 31.548 (3), 3.8219 (4)
*V* (Å^3^)	1562.6 (3)
*Z*	4
Radiation type	Mo *K*α
μ (mm^−1^)	0.11
Crystal size (mm)	0.51 × 0.36 × 0.29

Data collection	
Diffractometer	Bruker D8 Quest/Photon 100
Absorption correction	Multi-scan (*SADABS*; Bruker, 2014 ▸)
*T*_min_, *T*_max_	0.620, 0.746
No. of measured, independent and observed [*I* > 2σ(*I*)] reflections	16409, 1625, 1409
*R*_int_	0.044
(sin θ/λ)_max_ (Å^−1^)	0.625

Refinement	
*R*[*F* ^2^ > 2σ(*F* ^2^)], *wR*(*F* ^2^), *S*	0.040, 0.093, 1.16
No. of reflections	1625
No. of parameters	121
H-atom treatment	H-atom parameters constrained
Δρ_max_, Δρ_min_ (e Å^−3^)	0.22, −0.28

Computer programs: *APEX2*, *SAINT*, *XCIF* and *XPREP* (Bruker, 2014 ▸), *SHELXT2014/4* (Sheldrick, 2015*a* ▸), *SHELXL2014/6* (Sheldrick, 2015*b* ▸), *SHELXTL* (Sheldrick, 2008 ▸), *Mercury* (Macrae *et al.*, 2008 ▸) and *publCIF* (Westrip, 2010 ▸).

## supplementary crystallographic information

### Crystal data

**Table:**

C_20_H_14_O_6_	*D*_x_ = 1.489 Mg m^−^^3^
*M**_r_* = 350.31	Mo *K*α radiation, λ = 0.71073 Å
Orthorhombic, *P**n**m**a*	Cell parameters from 6581 reflections
*a* = 12.9597 (12) Å	θ = 2.6–28.1°
*b* = 31.548 (3) Å	µ = 0.11 mm^−^^1^
*c* = 3.8219 (4) Å	*T* = 100 K
*V* = 1562.6 (3) Å^3^	Prism, colorless
*Z* = 4	0.51 × 0.36 × 0.29 mm
*F*(000) = 728	

### Data collection

**Table:**

Bruker D8 Quest/Photon 100 diffractometer	1625 independent reflections
Radiation source: microfocus sealed tube	1409 reflections with *I* > 2σ(*I*)
Multilayer mirrors monochromator	*R*_int_ = 0.044
profile data from φ and ω scans	θ_max_ = 26.4°, θ_min_ = 2.6°
Absorption correction: multi-scan (SADABS; Bruker, 2014)	*h* = −16→16
*T*_min_ = 0.620, *T*_max_ = 0.746	*k* = −39→39
16409 measured reflections	*l* = −4→4

### Refinement

**Table:**

Refinement on *F*^2^	Primary atom site location: structure-invariant direct methods
Least-squares matrix: full	Secondary atom site location: difference Fourier map
*R*[*F*^2^ > 2σ(*F*^2^)] = 0.040	Hydrogen site location: inferred from neighbouring sites
*wR*(*F*^2^) = 0.093	H-atom parameters constrained
*S* = 1.16	*w* = 1/[σ^2^(*F*_o_^2^) + (0.0297*P*)^2^ + 1.0258*P*] where *P* = (*F*_o_^2^ + 2*F*_c_^2^)/3
1625 reflections	(Δ/σ)_max_ < 0.001
121 parameters	Δρ_max_ = 0.22 e Å^−^^3^
0 restraints	Δρ_min_ = −0.28 e Å^−^^3^

### Special details

**Table:**

Experimental. One distinct cell was identified using APEX2 (Bruker, 2014). Four frame series were integrated and filtered for statistical outliers using SAINT (Bruker, 2014) then corrected for absorption by integration using SAINT/SADABS v2014/2 (Bruker, 2014) to sort, merge, and scale the combined data. No decay correction was applied.
Geometry. All esds (except the esd in the dihedral angle between two l.s. planes) are estimated using the full covariance matrix. The cell esds are taken into account individually in the estimation of esds in distances, angles and torsion angles; correlations between esds in cell parameters are only used when they are defined by crystal symmetry. An approximate (isotropic) treatment of cell esds is used for estimating esds involving l.s. planes.
Refinement. Structure was phased by direct methods (Sheldrick, 2015). Systematic conditions suggested the ambiguous space group. The space group choice was confirmed by successful convergence of the full-matrix least-squares refinement on F^2^. The final map had no other significant features. A final analysis of variance between observed and calculated structure factors showed some dependence on amplitude and little dependence on resolution.

### Fractional atomic coordinates and isotropic or equivalent isotropic displacement parameters (Å^2^)

**Table:**

	*x*	*y*	*z*	*U*_iso_*/*U*_eq_	
O1	0.53843 (8)	0.37983 (3)	0.6378 (3)	0.0182 (3)	
O2	0.64043 (8)	0.32686 (3)	0.6752 (3)	0.0176 (3)	
O3	0.49910 (8)	0.32050 (3)	0.3282 (3)	0.0205 (3)	
C1	0.45214 (11)	0.40226 (5)	0.5132 (4)	0.0149 (3)	
C2	0.47199 (12)	0.44105 (5)	0.3612 (4)	0.0166 (3)	
H2	0.5410	0.4506	0.3305	0.020*	
C3	0.38982 (12)	0.46595 (5)	0.2539 (4)	0.0185 (3)	
H3	0.4022	0.4928	0.1494	0.022*	
C4	0.28937 (12)	0.45171 (5)	0.2990 (4)	0.0175 (3)	
H4	0.2331	0.4687	0.2238	0.021*	
C5	0.27117 (12)	0.41276 (5)	0.4532 (4)	0.0178 (3)	
H5	0.2023	0.4031	0.4839	0.021*	
C6	0.35262 (12)	0.38772 (5)	0.5632 (4)	0.0159 (3)	
H6	0.3404	0.3611	0.6710	0.019*	
C7	0.55226 (11)	0.33988 (5)	0.5243 (4)	0.0144 (3)	
C8	0.68026 (11)	0.28716 (5)	0.5719 (4)	0.0141 (3)	
C9	0.63026 (16)	0.2500	0.6630 (6)	0.0146 (4)	
H9	0.5657	0.2500	0.7808	0.018*	
C10	0.77487 (11)	0.28802 (5)	0.4077 (4)	0.0152 (3)	
H10	0.8068	0.3143	0.3513	0.018*	
C11	0.82269 (16)	0.2500	0.3264 (6)	0.0159 (5)	
H11	0.8882	0.2500	0.2149	0.019*	

### Atomic displacement parameters (Å^2^)

**Table:**

	*U*^11^	*U*^22^	*U*^33^	*U*^12^	*U*^13^	*U*^23^
O1	0.0145 (5)	0.0142 (5)	0.0258 (7)	0.0025 (4)	−0.0060 (5)	−0.0041 (5)
O2	0.0132 (5)	0.0147 (5)	0.0250 (6)	0.0033 (4)	−0.0045 (5)	−0.0041 (5)
O3	0.0155 (5)	0.0189 (6)	0.0272 (6)	0.0020 (4)	−0.0062 (5)	−0.0059 (5)
C1	0.0134 (7)	0.0153 (7)	0.0159 (8)	0.0029 (6)	−0.0022 (6)	−0.0035 (6)
C2	0.0142 (7)	0.0162 (8)	0.0193 (8)	−0.0034 (6)	0.0018 (6)	−0.0025 (6)
C3	0.0207 (8)	0.0151 (7)	0.0198 (8)	0.0005 (6)	0.0015 (7)	0.0000 (6)
C4	0.0153 (7)	0.0180 (8)	0.0192 (8)	0.0043 (6)	−0.0014 (6)	−0.0011 (7)
C5	0.0131 (7)	0.0217 (8)	0.0187 (8)	−0.0009 (6)	0.0020 (6)	−0.0035 (7)
C6	0.0176 (8)	0.0141 (7)	0.0161 (8)	−0.0014 (6)	0.0014 (6)	0.0002 (6)
C7	0.0110 (7)	0.0149 (7)	0.0173 (8)	−0.0002 (5)	0.0012 (6)	0.0010 (6)
C8	0.0139 (7)	0.0133 (8)	0.0151 (7)	0.0019 (6)	−0.0043 (6)	−0.0019 (6)
C9	0.0091 (10)	0.0175 (11)	0.0171 (11)	0.000	−0.0008 (8)	0.000
C10	0.0144 (7)	0.0162 (8)	0.0151 (7)	−0.0027 (6)	−0.0024 (6)	0.0010 (6)
C11	0.0116 (10)	0.0211 (11)	0.0150 (11)	0.000	−0.0002 (8)	0.000

### Geometric parameters (Å, º)

**Table:**

O1—C7	1.3446 (18)	C4—H4	0.9500
O1—C1	1.4064 (18)	C5—C6	1.384 (2)
O2—C7	1.3442 (18)	C5—H5	0.9500
O2—C8	1.4109 (18)	C6—H6	0.9500
O3—C7	1.1878 (18)	C8—C10	1.377 (2)
C1—C2	1.379 (2)	C8—C9	1.3842 (19)
C1—C6	1.382 (2)	C9—C8^i^	1.3841 (19)
C2—C3	1.385 (2)	C9—H9	0.9500
C2—H2	0.9500	C10—C11	1.3856 (18)
C3—C4	1.388 (2)	C10—H10	0.9500
C3—H3	0.9500	C11—C10^i^	1.3856 (18)
C4—C5	1.383 (2)	C11—H11	0.9500
			
C7—O1—C1	117.93 (12)	C1—C6—H6	120.6
C7—O2—C8	117.53 (12)	C5—C6—H6	120.6
C2—C1—C6	121.79 (14)	O3—C7—O2	127.33 (14)
C2—C1—O1	116.16 (13)	O3—C7—O1	127.50 (14)
C6—C1—O1	121.89 (14)	O2—C7—O1	105.16 (12)
C1—C2—C3	118.98 (14)	C10—C8—C9	123.23 (14)
C1—C2—H2	120.5	C10—C8—O2	115.82 (13)
C3—C2—H2	120.5	C9—C8—O2	120.67 (14)
C2—C3—C4	120.04 (15)	C8^i^—C9—C8	115.8 (2)
C2—C3—H3	120.0	C8^i^—C9—H9	122.1
C4—C3—H3	120.0	C8—C9—H9	122.1
C5—C4—C3	120.04 (14)	C8—C10—C11	118.90 (15)
C5—C4—H4	120.0	C8—C10—H10	120.5
C3—C4—H4	120.0	C11—C10—H10	120.5
C4—C5—C6	120.43 (14)	C10—C11—C10^i^	119.9 (2)
C4—C5—H5	119.8	C10—C11—H11	120.0
C6—C5—H5	119.8	C10^i^—C11—H11	120.0
C1—C6—C5	118.71 (14)		
			
C7—O1—C1—C2	−125.86 (15)	C8—O2—C7—O1	−173.24 (12)
C7—O1—C1—C6	58.7 (2)	C1—O1—C7—O3	−0.4 (2)
C6—C1—C2—C3	−0.4 (2)	C1—O1—C7—O2	178.35 (12)
O1—C1—C2—C3	−175.82 (14)	C7—O2—C8—C10	116.32 (15)
C1—C2—C3—C4	−0.2 (2)	C7—O2—C8—C9	−69.5 (2)
C2—C3—C4—C5	0.4 (2)	C10—C8—C9—C8^i^	−1.4 (3)
C3—C4—C5—C6	−0.1 (2)	O2—C8—C9—C8^i^	−175.11 (11)
C2—C1—C6—C5	0.8 (2)	C9—C8—C10—C11	0.5 (3)
O1—C1—C6—C5	175.92 (14)	O2—C8—C10—C11	174.47 (15)
C4—C5—C6—C1	−0.5 (2)	C8—C10—C11—C10^i^	0.5 (3)
C8—O2—C7—O3	5.6 (2)		

Symmetry code: (i) *x*, −*y*+1/2, *z*.

### Hydrogen-bond geometry (Å, º)

**Table:**

*D*—H···*A*	*D*—H	H···*A*	*D*···*A*	*D*—H···*A*
C10—H10···O3^ii^	0.95	2.59	3.2105 (8)	123

Symmetry code: (ii) *x*+1/2, *y*, −*z*+1/2.
